# Serum metabolomic profiling predicts synovial gene expression in rheumatoid arthritis

**DOI:** 10.1186/s13075-018-1655-3

**Published:** 2018-08-03

**Authors:** Rekha Narasimhan, Roxana Coras, Sara B. Rosenthal, Shannon R. Sweeney, Alessia Lodi, Stefano Tiziani, David Boyle, Arthur Kavanaugh, Monica Guma

**Affiliations:** 1 Division of Rheumatology, University of California, San Diego, 9500 Gilman Drive, San Diego, CA 92093 USA; 2 Department of Medicine, Autonomous University of Barcelona, Plaça Cívica, 08193 Bellaterra, Barcelona, Spain; 3 Center for Computational Biology and Bioinformatics, University of California, San Diego, 9500 Gilman Drive, San Diego, CA 92093 USA; 4 Department of Nutritional Sciences & Dell Pediatric Research Institute, Dell Medical School, University of Texas at Austin, 1400 Barbara Jordan Blvd, Austin, TX USA

**Keywords:** NMR, Metabolomics, Biomarkers, Synovium, Rheumatoid arthritis, Gene expression

## Abstract

**Background:**

Metabolomics is an emerging field of biomedical research that may offer a better understanding of the mechanisms of underlying conditions including inflammatory arthritis. Perturbations caused by inflamed synovial tissue can lead to correlated changes in concentrations of certain metabolites in the synovium and thereby function as potential biomarkers in blood. Here, we explore the hypothesis of whether characterization of patients’ metabolomic profiles in blood, utilizing ^1^H-nuclear magnetic resonance (NMR), predicts synovial marker profiling in rheumatoid arthritis (RA).

**Methods:**

Nineteen active, seropositive patients with RA, on concomitant methotrexate, were studied. One of the involved joints was a knee or a wrist appropriate for arthroscopy. A Bruker Avance 700 MHz spectrometer was used to acquire NMR spectra of serum samples. Gene expression in synovial tissue obtained by arthroscopy was analyzed by real-time PCR. Data processing and statistical analysis were performed in Python and SPSS.

**Results:**

Analysis of the relationships between each synovial marker-metabolite pair using linear regression and controlling for age and gender revealed significant clustering within the data. We observed an association of serine/glycine/phenylalanine metabolism and aminoacyl-tRNA biosynthesis with lymphoid cell gene signature. Alanine/aspartate/glutamate metabolism and choline-derived metabolites correlated with TNF-α synovial expression. Circulating ketone bodies were associated with gene expression of synovial metalloproteinases. Discriminant analysis identified serum metabolites that classified patients according to their synovial marker levels.

**Conclusion:**

The relationship between serum metabolite profiles and synovial biomarker profiling suggests that NMR may be a promising tool for predicting specific pathogenic pathways in the inflamed synovium of patients with RA.

**Electronic supplementary material:**

The online version of this article (10.1186/s13075-018-1655-3) contains supplementary material, which is available to authorized users.

## Background

The hallmark of rheumatoid arthritis (RA) is chronic synovitis that affects multiple joints and invades cartilage causing bone erosions and joint destruction [1]. As the synovium is the principal target of inflammation in RA, and the resident synoviocytes (fibroblast-like synoviocytes and macrophages-like synoviocytes) along with recruited cells (myeloid cells and lymphocytes) are implicated in the pathogenesis of synovitis, special interest has been given to the study of synovial tissue in this disease. These studies not only aim to clarify RA pathogenesis and provide insight into the mechanisms of action of therapeutic interventions [1, 2], but are also a promising approach to search for biomarkers in the inflamed synovial tissue [1]. Changes in the cellular infiltrate or biomarkers such as cytokines or growth factors in RA-affected synovial tissue have long been known to be associated with the clinical course of disease and have been used to identify specific responses to RA therapies [1–4]. Recently there has been increasing interest in synovial biopsies to obtain inflamed synovial tissue from joints and thereby gain a better understanding of the pathogenic events in these diseases [5]. Histopathotype and pathological pathways-based patient stratification prior to therapeutic intervention could be exploited to identify biomarker predictors of clinical outcomes and responses to therapy [6, 7].

Tissue pathology and pathogenic pathways cannot yet be reliably explored through noninvasive circulating or imaging biomarkers. Given the complexity and heterogeneous nature of RA, it is unlikely that a single cytokine will provide sufficient discrimination between patients and thus be a good biomarker [8, 9]. Global biomarker signatures may represent a more appropriate approach for improving treatment protocols and outcomes for patients with RA. Metabolomics is the science of identifying and quantifying the biochemical byproducts of metabolism in a cell, tissue, or organism [10]. Metabolomics is an emerging field of biomedical research that can offer a better understanding of the mechanisms underlying disease and help to develop new strategies for treatment [11]. Unlike genes and proteins, which are epigenetically regulated and post-translationally modified, metabolites are direct signatures of biochemical activity and thus it may be easier to test whether they are correlated with phenotype [12].

The fundamental rationale in metabolomics is that perturbations caused by a disease in a biological system will lead to changes that are correlated with the concentrations of certain metabolites [13, 14]. Metabolite patterns represent the final response of biological systems to disease status, or in response to a medical or external intervention [12]. ^1^H-nuclear magnetic resonance (NMR) can delineate patterns of changes in biomarkers that are highly discriminatory for the observed disease or intervention [15]. We propose in this work, that the study of metabolomics in serum from patients with RA, using NMR, can be used to predict synovial pathology. We hypothesize that perturbations caused by inflamed synovial tissue will lead to changes that correlate with the concentration of certain metabolites in the synovium. These changes will then be reflected in blood serum and function as potential biomarkers of different synovial markers. Here we describe the first study that defines metabolite signatures in serum that correlate with gene expression profiling in synovial tissue from patients with active RA.

## Methods

### Patients

The Assessment of rituximab’s immunomodulatory synovial effects (ARISE) clinical trial (registered at ClinicalTrials.gov, NCT00147966) has been described in detail [3]. Briefly, the study enrolled people between 18 and 70 years of age with an established diagnosis of RA and a positive serum test for rheumatoid factor (RF). Patients had to have active disease (defined as a tender joint count > 8/68, a swollen joint count > 6/66, and either early morning stiffness > 45 min in duration, or an elevation in erythrocyte sedimentation rate (ESR) > 28 mm/h or C-reactive protein (CRP) > 1.5 mg/dL), despite the concomitant use of methotrexate (MTX) at a dose of > 12.5 mg/week for at least 12 weeks. One of the involved joints had to be a knee or a wrist that could be appropriately examined by arthroscopy. Concomitant use of non-steroidal anti-inflammatory drugs and oral prednisone at doses of 10 mg/day or less were permitted, provided dosing was stable for at least 4 weeks before the study. Patients previously treated with tumor necrosis factor (TNF-α) inhibitors were permitted to enroll in the study provided they had been off therapy for > 2 months for etanercept and > 3 months for adalimumab or infliximab. Patients meeting eligibility criteria underwent baseline arthroscopic synovial biopsy of an affected knee or wrist. Nineteen patients for whom both baseline synovial biopsy gene expression data and baseline serum metabolomics data were available were analyzed in the current study. Clinical disease parameters, including disease activity score (DAS), health assessment questionnaire (HAQ), pain, joint swelling and tenderness, ESR, RF, and anti-cyclic citrullinated peptide (anti-CCP) are described in Additional file 1: Table S1.

### Synovial gene expression analysis

Synovial RNA was extracted from pools of six tissue fragments and complementary DNA (cDNA) was synthesized. TaqMan PCR was performed using predeveloped reagents (Applied Biosystems, Foster City, CA, USA) as described previously [3]. Gene expression, utilizing quantitative reverse transcriptase (RT)-PCR, was performed to measure inflammatory mediators and B cell survival factors, including IgM (heavy chain), IgG (heavy chain), IgKappa (light chain), CD3E, TNF-α, interleukin (IL)-1β, IL-6, IL-8, matrix metalloproteinase (MMP)-1, MMP-3, B lymphocyte stimulator (BLyS), stromal cell-derived factor 1 (SDF1), and a proliferation-inducing ligand (APRIL). Synovial gene expression for the 19 patients analyzed in this study are summarized in Additional file 1: Table S2.

### ELISA

TNF-α, MMP3 IL-1β, and IL-6 from serum were evaluated by DuoSet enzyme-linked immunosorbent assays following the manufacturer’s protocol (R&D systems).

### Metabolomics analysis

Frozen serum was obtained from the Division of Rheumatology, Allergy, and Immunology at the University of California (UC) San Diego School of Medicine (San Diego, CA, USA). Lipid and protein fractions were removed via ultrafiltration (Nanosep 3 K OMEGA, Pall Corporation, Ann Arbor, MI, USA) at 4 °C. The filtered biofluid was used for NMR analysis. An aliquot of 160 μL of filtered serum was mixed with 20 μL D_2_O and 20 μL of phosphate buffer (100 mM final concentration) containing TMSP-d4 (0.1 mM final concentration) and sodium azide (0.05% (*w*/*v*) final concentration). The prepared samples were centrifuged to remove any remaining particulates and a 180 μL aliquot was transferred to a 3 mm NMR tube (Norell, Landisville, NJ, USA) prior to acquisition. NMR spectra were acquired with a 16.4 T (700 MHz) Bruker Avance spectrometer (Bruker BioSpin Corp., Billerica, MA, USA) equipped with a 5 mm TCI cryogenically cooled probe and autosampler at 30 °C. Following acquisition, spectra were processed using NMRlab and MetaboLab [16]. Metabolite assignment and quantification was performed using several databases [16]. Metabolite assignment and quantification was performed using Chenomx NMR Suite (Chenomx Inc., Edmonton, AB, Canada), the Birmingham Metabolite Library [17], and the Human Metabolome Database [18]. The NMR results were recently published [19] and are summarized in Additional file 1: Table S3 for the 19 patients analyzed in this study.

### Data analysis

The data, consisting of 19 patient samples measured across 18 synovial markers and 49 metabolites, were processed using Python. Hierarchically clustered heatmaps were generated for correlation between synovial markers and metabolites separately. Hierarchical clustering and visualization was performed using the scientific computing package SciPy, and the visualization package Seaborn (https://seaborn.pydata.org/). Dendrograms were divided into flat clusters using a cophenetic distance metric. Linear regression was performed between each cytokine-metabolite pair, controlling for patient age and gender using the ordinary least squares (OLS) method from the Python package StatsModels. Normally distributed independent variables were standardized so they had a mean of zero and a standard deviation of one. Discriminant analyses were performed to determine coefficients for linear combinations of variables that assigned cluster membership to individual cases. Basic descriptive statistics used to describe the patient population and discriminant analysis were performed using the SPSS software version 15.0.

## Results

### Synovial marker and blood metabolite clustering

**Fig. 1:**
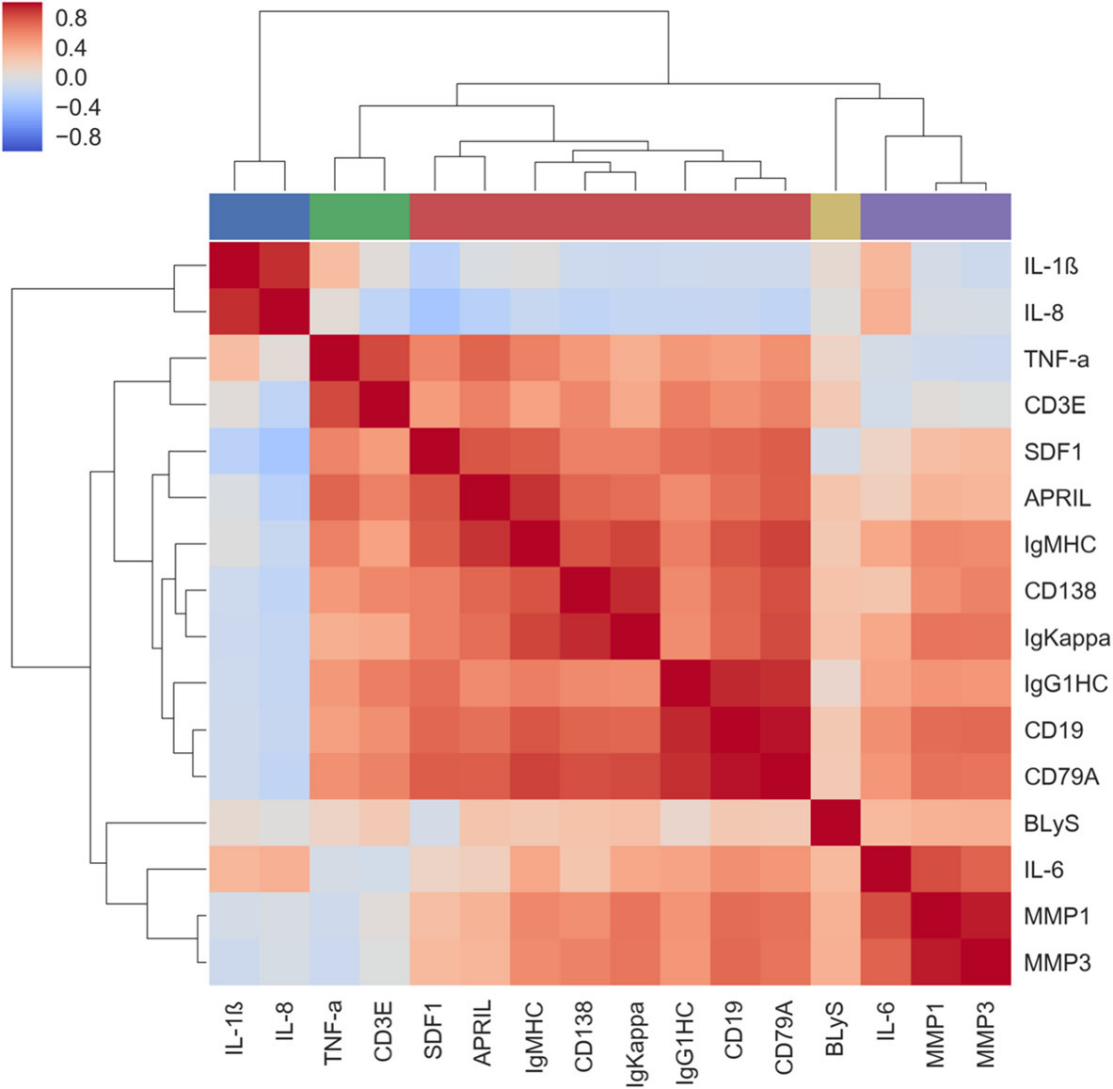
Synovial markers clustering. Heat map and hierarchical cluster analysis indicates positive relationships between cytokines identified by quantitative PCR in synovial tissue from patients with rheumatoid arthritis. Pearson’s correlation coefficients for each metabolite and hierarchical clustering with Euclidean distance metric are included. The color bar along the top indicates cytokine grouping based on hierarchical clustering. APRIL, a proliferation-inducing ligand; BLyS, lymphocyte stimulator; MMP, matrix metalloproteinase; SDF1, S cell-derived factor 1

We first analyzed whether synovial markers clustered into different groups (Fig. 1). IL-6, MMP1, and MMP3 are strongly correlated among themselves but are inversely correlated with TNF-α, which interestingly, is strongly correlated with CD3E. MMP1 and MMP3 are also inversely correlated with another cluster that includes IL-1β and IL-8. In addition, there was a big cluster comprising B and plasma cell markers, and growth factors, including SDF1, APRIL, CD138, CD19, CD79A, IgG and IgM heavy chains, and IgKappa.

**Fig. 2:**
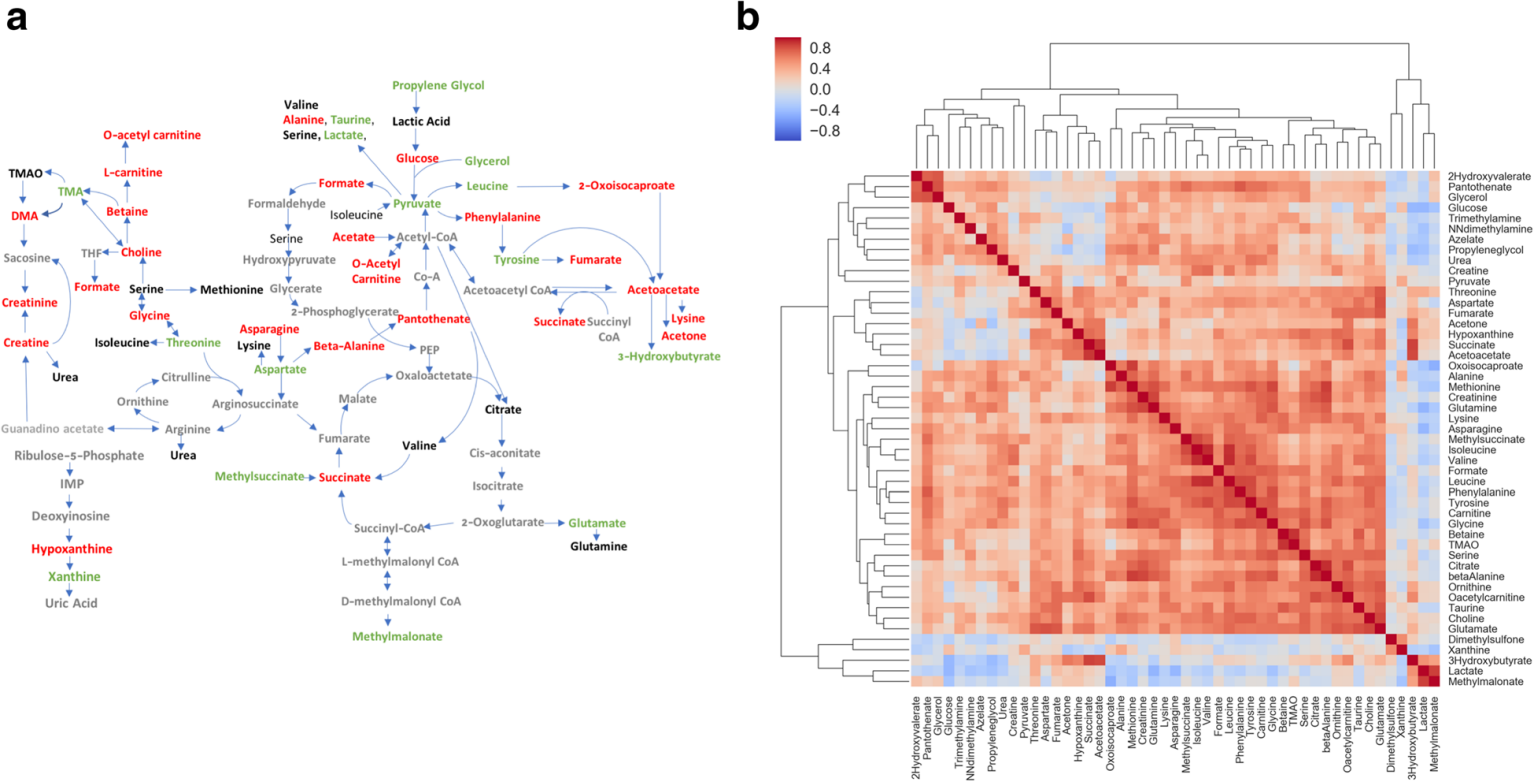
Blood metabolite clustering. **a** Overview of the metabolites identified by ^1^H-nuclear magnetic resonance (NMR) organized by metabolic pathway. Metabolites that were elevated by at least 20% compared to reference values are in green and metabolites that were decreased by more than 20% compared to reference values are in red.. Metabolites not identified by NMR are in gray. Abbreviations: TMA, trimethylamine; TMAO, trimethylamine N-oxide; DMA, NN-dimethylamine; THF, tetrahydrofolate; IMP, inosine monophosphate. **b** Heat map and hierarchical cluster analysis indicate positive relationships between polar metabolites identified by ^1^H-NMR in serum from patients with rheumatoid arthritis before treatment with rituximab. Pearson’s correlation coefficients for each metabolite and hierarchical clustering with Euclidean distance metric are shown

We also characterized the blood metabolites. As shown in Additional file 1: Table S3 and Fig. 2a, most of the metabolites were downregulated compared to reference values, suggesting that these metabolites might be consumed by the inflamed synovium due to an increase in its metabolic demand. A few metabolites were upregulated compared to reference values, including glycolytic metabolites such as lactate and pyruvate. This likely reflects the increased bioenergetic and biosynthetic demands of sustained inflammation. Choline metabolism has recently been strongly related to inflammation [20]. Dietary intake of choline, through two circulating metabolites, trimethylamine (TMA) and trimethylamine N-oxide (TMAO), are mechanistically linked to cardiovascular inflammation [20]. Interestingly TMA was also elevated in our patients with RA. In addition, 3-hydroxybutyrate, a ketone body, and select amino acids, such as leucine, threonine, tyrosine, and aspartate were upregulated in patients with active RA. We also analyzed whether blood metabolites could be clustered in groups. Metabolites primarily clustered into groups according to their biological function or chemical classification (Fig. 2b). As expected, the group of metabolites that were elevated in patients, namely lactate, methylmalonate, xanthine, and 3-hydroxybutyrate, were inversely correlated with the most of metabolites.

### Linear regression analysis between grouped synovial markers and metabolites

**Fig. 3:**
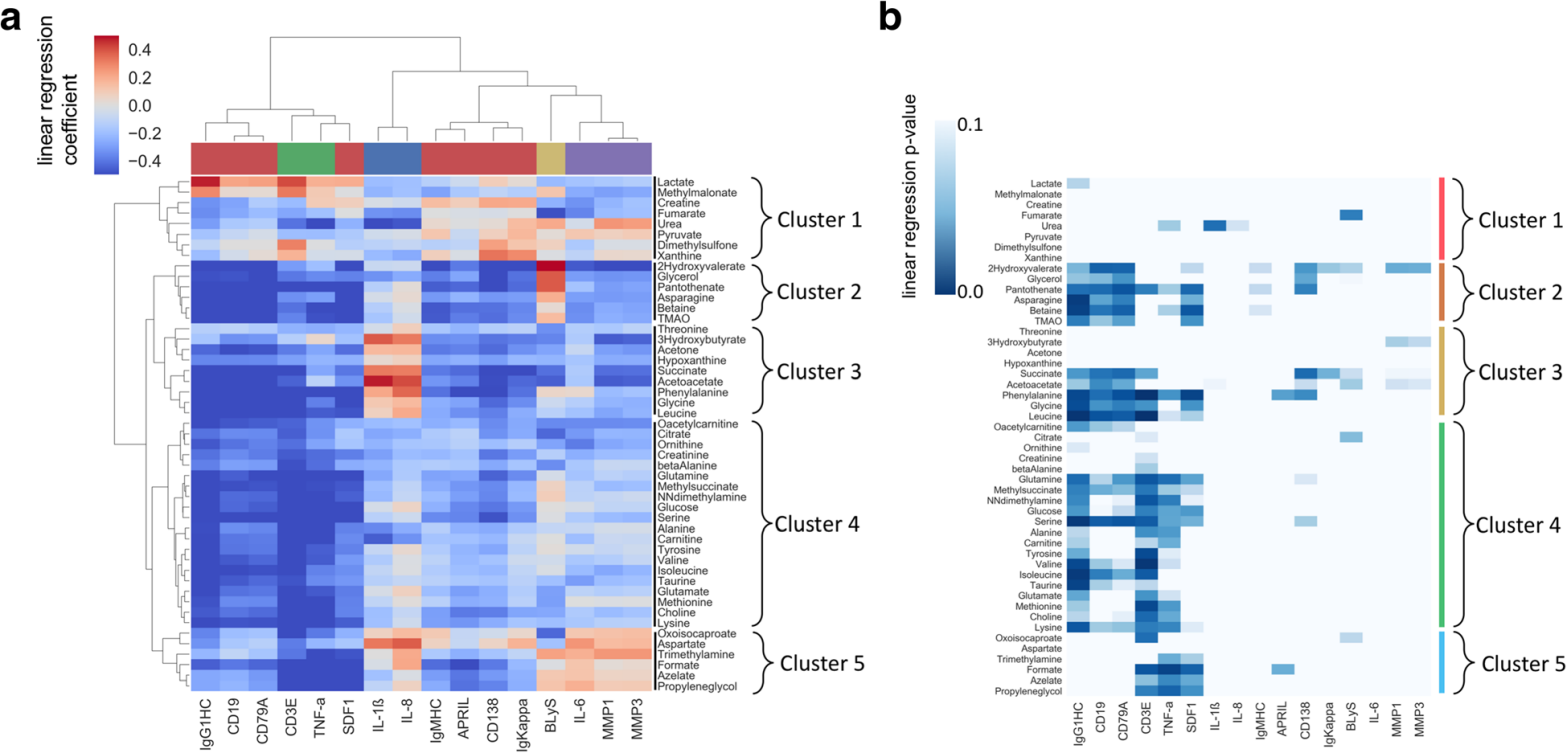
Correlation of synovial markers with serum metabolites. **a** Linear regression was performed between each synovial marker–serum metabolite pair, controlling for age and gender. The regression coefficients for each pair were used to form a clustered heatmap, to lend insight into which groups of synovial markers were correlated with which groups of metabolites. The color bar along the top is preserved from Fig. 1, and indicates groups of similar cytokines. Row clusters have been identified by cophenetic cutting of the row dendrogram. **b** Metabolite regression *p* values are displayed in Fig. 3b, where the row and column order are preserved from Fig. 3a. APRIL, a proliferation-inducing ligand; BLyS, lymphocyte stimulator; MMP, matrix metalloproteinase; SDF1, S cell-derived factor 1

Linear regression was analyzed between each synovial marker-metabolite pair; age and gender were controlled for by including these factors as covariates in the model. The regression coefficients for each cytokine-metabolite pair were used to form a clustered heatmap to lend insight into which groups of synovial markers were correlated with which groups of metabolites. We observed significant clustering structures in the data (Fig. 3a). The color bar along the top of Fig. 3 preserves the synovial marker clusters from Fig. 1. Interestingly the clusters of synovial markers almost correspond to the clusters observed within synovial markers (Fig. 1), suggesting that cytokine clusters in the synovial tissue have a similar metabolite signature in blood. The most striking difference is seen in the cluster comprising SDF1, APRIL, CD138, CD19, CD79A, and IgG and IgM heavy chain, and IgKappa in Fig. 1, which is seen split into two groups in Fig. 3. One metabolite signature correlates with CD19, CD79A and IgG heavy chain, markers of B cells; and the other metabolite signature correlates with SDF1, APRIL, CD138 and IgM heavy chain, markers related to plasma cell biology. Of interest BLyS, had a different metabolite profile than the rest of plasma cell biomarkers. Metabolite regression *p* values are displayed in Fig. 3b, where the row and column order are preserved from Fig. 3a and Additional file 2: Figure S1.

**Fig. 4:**
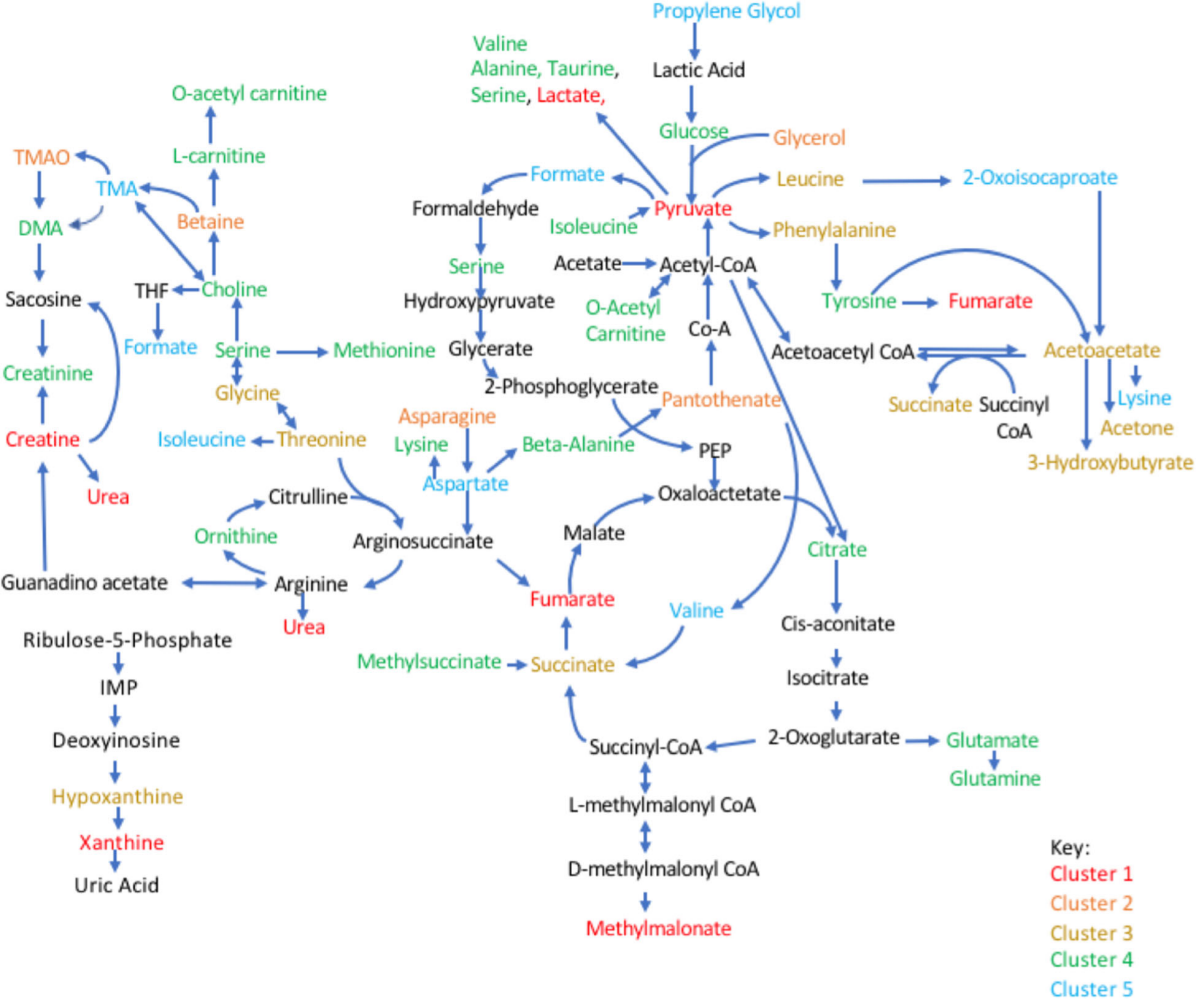
Identified metabolites clusters. Overview of the metabolites identified by ^1^H-nuclear magnetic resonance organized by metabolic pathway and colored by cluster. Abbreviations: TMA, trimethylamine; TMAO, trimethylamine N-oxide; DMA, NN-dimethylamine; THF, tetrahydrofolate; IMP, inosine monophosphate

As observed in Fig. 3, metabolites can be grouped into five clusters (Fig. 4) that were further analyzed using the MetaboAnalyst [21, 22] web tool for functional enrichment of these groups of metabolites. Both pathway significance and pathway impact were assessed using this tool (Additional file 3: Figure S2).

**Fig. 5:**
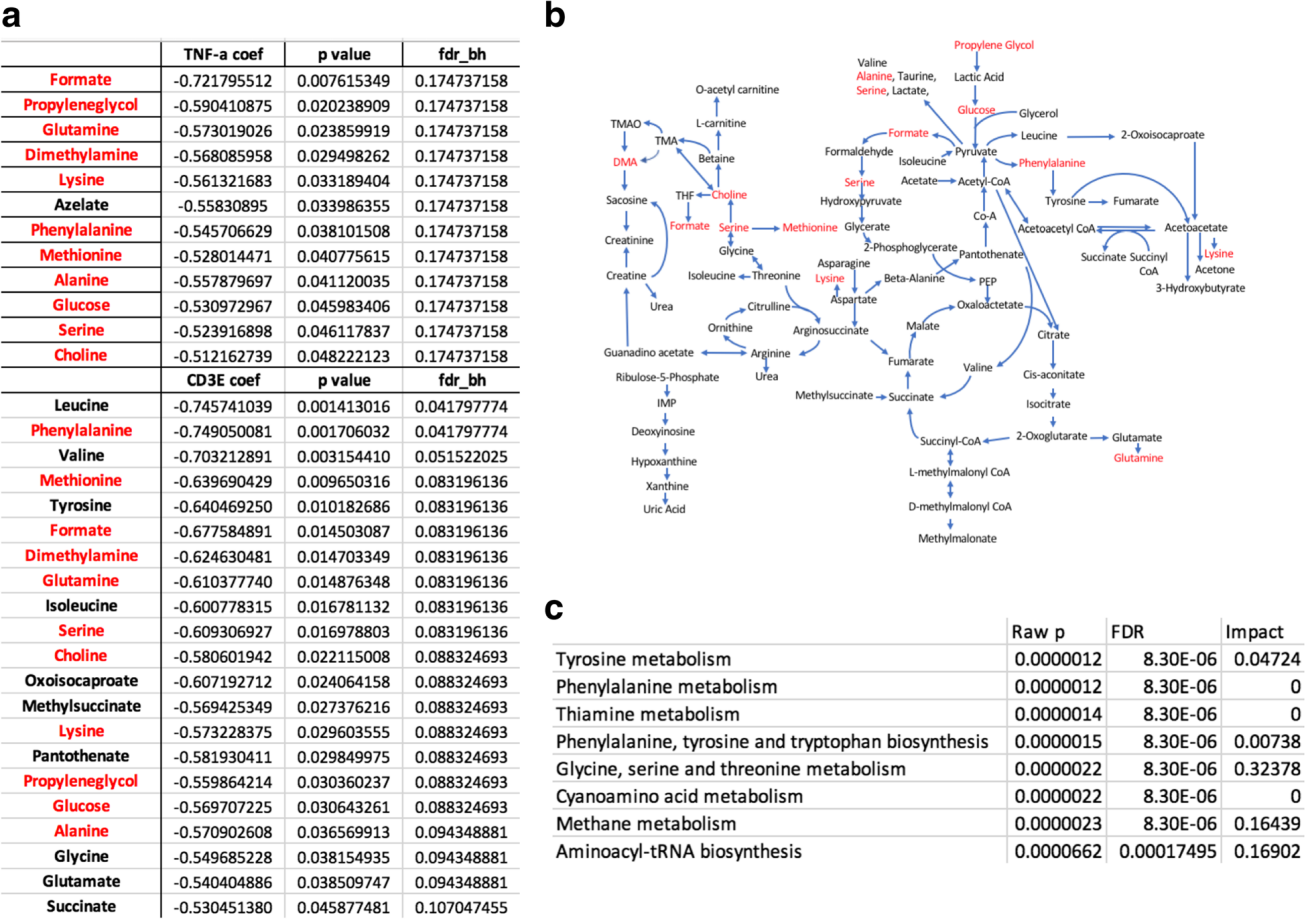
Correlation between serum metabolites and synovial TNF-α and CD3E. **a** Correlation between serum metabolites and each synovial marker, using linear regression, controlling for both age and gender. We also included *p* values adjusted for Benjamini-Hochberg false discovery rate (fdr_bh) to correct for multiple testing. **b** Overview of the metabolites identified by ^1^H-nuclear magnetic resonance organized by metabolic pathway. Metabolites that were negatively correlated with TNF-α and CD3 are shown in red. Abbreviations: TMA, trimethylamine; TMAO, trimethylamine N-oxide; DMA, NN-dimethylamine; THF, tetrahydrofolate; IMP, inosine monophosphate. **c** Pathway analysis of polar compounds by MetaboAnalyst. Pathway *p* values were calculated based on metabolites that were correlated with both TNF-α and CD3E. Coef, coefficient

We then determined the most strongly correlated or anti-correlated serum metabolites for each synovial marker, using linear regression, and controlling for both age and gender. We also included Benjamini-Hochberg false discovery rate (FDR)-adjusted *p* values to correct for multiple testing. As shown in Fig. 5, the synovial markers TNF-α and CD3E were negatively correlated with several metabolites in serum. The significant polar metabolites were mapped to known metabolic pathways using MetaboAnalyst 3.0 [22, 23]. and ranked by their overall *p* values (Fig. 5c). Additional file 4: Figure S3, Additional file 5: Figure S4, Additional file 6: Figure S5, and Additional file 7: Figure S6 show correlation between metabolites and the remaining synovial marker clusters.

### Discriminant analysis

**Fig. 6:**
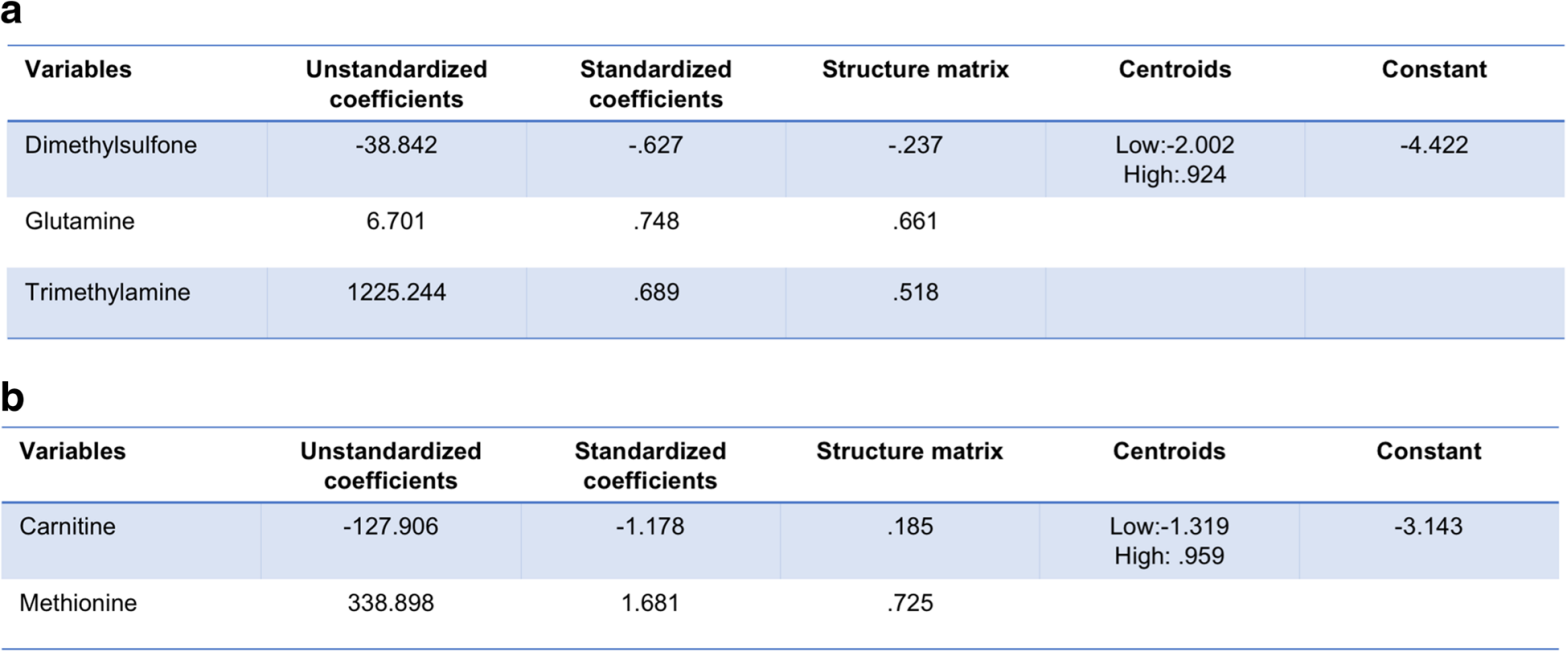
Discriminant analysis. Unstandardized and standardized discriminant function coefficients, structure matrix, centroids, and constant for direct discriminant function for TNF-α (**a**) and CD3E (**b**)

We then explored whether or not one or more metabolites in serum could discriminate between high or low levels of synovial marker gene expression. At present, no factors have been identified that fully explain or predict response to RA therapy [24], but pre-treatment differences at baseline between patient groups have been identified, including synovial tissue TNF expression and an increased number of synovial macrophages and T cells in patients who subsequently exhibited clinical improvement after initiation of anti-TNF therapy [25]. Therefore, we used stepwise discriminant function analyses to discriminate TNF-α or CD3E levels. Multivariate and cross-validation classification using the “leave-one-out” classification method was used for these calculations. We defined high or low marker levels according to their synovial marker gene expression mean. This stepwise discriminant analysis is presented in Fig. 6. For TNF-α discriminant analysis, three metabolites namely glutamine, TMA, and dimethylsulfone were sufficient to correctly classify 94.7% of TNF-α levels. There was canonical correlation of 0.821 and Wilks’ lambda of 0.326 when these three variables were used, with high significance (*p* < 0.001; Fig. 6a). For CD3E discriminant analysis, two metabolites namely carnitine and methionine were sufficient to correctly classify 89.6% of CD3E levels. There was canonical correlation of 0.765 and Wilks’ lambda of 0.414 when these three variables were used, with high significance (*p* < 0.001; Fig. 6b).

## Discussion

Our increasing understanding of the pathogenesis of RA has transformed the therapeutic options available for people with this disease. The introduction of newer agents and novel treatment strategies has resulted in improved outcomes for patients. However, these successes have raised the bar for the goals of therapy. At present, disease remission, or low disease activity at the very least, has become the new goal of treatment for all patients. Therefore, there is still an unmet need in RA. Biomarkers employed in “personalized” medicine might be useful in an attempt to match a patient with the most appropriate biologic therapy, and thereby optimize outcomes. The accessibility of a biological biomarker is an important factor in this approach [8]. Although sampling inflamed synovial tissue from joints might be critical to gain a better understanding of the pathogenic events of inflammatory arthritis, a biomarker that can be obtained in a minimally invasive manner is more attractive, particularly for patients in early stages of the disease, where mostly small joints are involved [8]. In this study, we attempt, for the first time, to find serum metabolomics profiles that correlate with synovial marker gene expression.

Recent studies have indicated that metabolic regulation and cell signaling are tightly and ubiquitously linked with immune responses. Metabolomics studies that aim to improve biological understanding through the analysis of metabolite profiles of the underlying biological pathways are certainly relevant and have been successful in other fields, especially oncology. Though the application of metabolomics to RA is still in its infancy, early studies have yielded promising results [19, 26–33]. A small number of metabolomics studies have focused on identifying metabolites associated with rheumatic diseases, primarily in the serum for diagnostic purposes [30–32], but none have attempted to predict synovial pathology.

We hypothesized that perturbations caused by inflamed synovial tissue will lead to changes that correlate with the concentrations of certain metabolites in the synovium that will be then reflected in blood serum. A recent publication on a study of metabolic profiling in the synovial tissue reported altered glucose and choline metabolism [34]. Both pathways have recently been involved in RA pathogenesis [27, 34, 35]. Choline levels in patients from our cohorts are decreased in blood compared to the normal range; this, along with an increased uptake in the joints on choline C-11 PET scanning in inflammatory arthritis [36] and high expression in fibrocyte-like synoviocytes (FLS) of choline like transporter (CTL)1 (high-affinity) and CTL2 (low-affinity) [37], suggest increased circulating choline uptake and consumption by the inflamed synovium. Glucose levels were decreased, and lactate levels increased in serum from our cohort. Glucose is consumed through upregulation of aerobic glycolysis and when metabolized, gives rise to production of copious amounts of lactate, which must be extruded from the cell to prevent lactic acidosis [38]. Several studies have highlighted the increase in glucose metabolism in the hypoxic joint [27, 35]. Thus, our results in serum seem to agree well with recently described synovial studies [34]. Of interest, both choline and glucose levels in the blood negatively correlated with TNF-α and CD3E gene expression in the synovium.

Literature in the field of oncology can help us to interpret some of our results. For instance, we observed an association of serine/glycine metabolism and aminoacyl-tRNA biosynthesis with TNF-α/CD3E and B/plasma cell signatures that suggest that lymphoid cells could be using these pathways after activation in the rheumatoid synovium. Although alterations in glucose and glutamine metabolism are central to metabolic transformation, recent studies have focused on the role of the nonessential amino acids serine and glycine in supporting tumor growth [39]. In addition to their role in protein synthesis, serine and glycine contribute to anabolic pathways important for the generation of glutathione, nucleotides, phospholipids, and other metabolites [40]. The requirement for intracellular serine and glycine for the support of cell growth and proliferation is clear. Other amino acids are also critical substrates that fuel mitochondrial metabolism and the biosynthesis of proteins, lipids, and other molecules. Of particular interest in cancer are key mitochondrial enzymes in the metabolism of glutamine, glutamate, proline, aspartate, and alanine [41]. The branched chain amino acids (BCAAs) valine, leucine, and isoleucine are also highly metabolized by transaminases. By coordinating cellular bioenergetics and biosynthesis through the tricarboxylic acid (TCA) cycle, amino acid metabolism could be critical not only in tumor cells but also in lymphoid cell proliferation and survival as described recently [42].

Another metabolite that correlates with several of our cytokine pathways is succinate. Succinate is an intermediate of the TCA cycle and plays a crucial role in adenosine triphosphate (ATP) generation in mitochondria. Recently, new roles for succinate outside metabolism have emerged. Succinate promotes expression of the pro-inflammatory cytokine IL-1β by inhibiting prolyl hydroxylases and stabilizing the transcription factor hypoxia-inducible factor-1α (HIF-1α) in activated macrophages, and stimulates dendritic cells via succinate receptor 1 [38, 43]. Furthermore, succinate has been shown to post-translationally modify proteins. Of interest, the succinate level in blood positively associated with synovial IL-1β gene expression although it did not reach statistical significance.

The cluster comprising MMP1/MMP3/IL-6, which could represent a fibroblast-driven phenotype, was negatively correlated with ketone bodies. Acetoacetate is the common precursor of the two other circulating ketone bodies, acetone and 3-hydroxybutyrate [44]. 3-hydroxybutyrate is the most abundant circulating ketone body and is less likely to degrade spontaneously into acetone than acetoacetate. One can speculate that rheumatoid fibroblasts require intracellular ketone bodies for the support of their invasive phenotype and that the increase in 3-hydroxybutyrate uptake and/or enzymes in this pathway could explain the negative correlation. Of note, the positive correlation between 3-hydroxybutyrate and IL-1β and IL-8 is also of interest, as 3-hydroxybutyrate, long viewed as a simple carrier of energy from the liver to peripheral tissues, also possesses signaling activities and is also an endogenous inhibitor of histone deacetylases (HDACs) [45]. Moreover, recent research has shown that 3-hydroxybutyrate can block the NOD-like receptor pyrin containing 3 (NLRP3) inflammasome [46]. Further studies are needed to understand the effect of these metabolites in the synovium in RA.

As mentioned above, metabolites can not only be biomarkers of perturbations caused by inflamed synovial tissue but also can have a pathogenic effect that would amplify synovial inflammation. Secondary roles have emerged for glucose metabolites, metabolic enzymes, and TCA cycle intermediates outside of metabolism. Not only succinate but also other metabolites including α-ketoglutarate, fumarate, and acetyl-CoA might be expected to accumulate in macrophages and FLS under hypoxic conditions, and are involved in eliciting important epigenetic changes, with unexplored potential for driving chronic inflammation [47, 48]. Also, essential glycolytic enzymes have been shown to translocate to the nucleus or mitochondria where they function independently of their canonical metabolic roles in the regulation of cytokines and anti-apoptotic responses [49, 50]. Thus, metabolomics studies have also the potential of defining the elements of synovial metabolic pathobiology.

Although NMR spectroscopy has less sensitivity compared to mass spectrometry instrumentation, NMR requires minimal sample preparation, and is not only non-destructive, inherently untargeted, highly reproducible [51, 52], and intrinsically quantitative, but is also cheaper and more accessible than mass spectrometry [53–55]. Depending on the biological samples, NMR can identify and quantify more than 200 metabolites in an untargeted fashion and more than 100 metabolites are uniquely identified by NMR [56]. In this work, we also showed that the combination of only two or three metabolites identified in serum by NMR could discriminate between high or low levels of synovial TNF-α and CD3E gene expression. Studies in other cohorts of patients with active RA are needed to validate these results, yet the relationship between serum metabolic profiles and synovial biomarker profiling suggests that NMR may be a promising tool for predicting specific pathogenic pathways in the inflamed synovium in RA.

Although these findings are certainly promising, this study is not without limitations. Most importantly, we evaluated a small number of clinical samples. Despite similar clinical parameters for patient inclusion, large biological variance is expected in primary samples. In addition, patients had long-standing disease and were exposed to various therapies prior to the study, and were on methotrexate at the time of the study, which is reported to change several metabolic pathways including adenosine metabolism [57]. Confirmation of our results in a larger sample size from a cohort of patients with new onset inflammatory arthritis before treatment initiation, studied prospectively, is necessary to strengthen our conclusions. Comparison with other arthritides or other systemic inflammatory diseases to determine if these changes in metabolite levels come from the joints or from different sources is also critical to interpret our results. One other confounder is the microbiome, which is altered in RA and can potentially cause metabolic changes in both serum and synovial tissues [58–60]. In addition, further studies are needed to evaluate the relationship between circulating metabolites and synovial pathology. Metabolite profiles in blood, if they correlate with metabolic changes in synovial tissue, will certainly reveal more about RA etiology. We did not identify correlation between cytokine serum levels and cytokine synovial gene expression (Additional file 8: Figure S7), yet it remains unknown whether or not metabolic changes will display stronger correlation between blood and synovium.

## Conclusions

The relationship between serum metabolite profiles and synovial biomarker profiling suggests that NMR may be a promising tool for predicting specific pathogenic pathways in the inflamed synovium of patients with RA. Further studies will help to better test the correlation and understand the metabolic profiles between cytokine and cell signatures, and address whether or not NMR metabolomics can be used to stratify patients with RA by predicting specific cellular infiltrates or other synovial biomarkers, and to identify specific responses to RA therapies.

## Additional files

Additional file 1: **Table S1.** Baseline clinical characteristics of patients with rheumatoid arthritis. **Table S2.** Mean and standard deviation (SD) of synovial biomarker expression. **Table S3.** Mean and standard deviation (SD) of serum metabolites detected by ^1^H-NMR (μM). Reference values are from the Human Metabolome Database (HMDB) and were collected via NMR, unless otherwise noted. ^1^GC/MS; ^2^HPLC; ^3^HPLC-fluoroescence; ^4^ion-exchange chromatography; ^5^DFI/MS/MS ^6^unknown. ND, no data available. Metabolites that were upregulated by at least 20% compared to reference values are in green. Metabolites that were downregulated by more than 20% compared to reference values are in red. (DOCX 26 kb)

Additional file 2: **Figure S1.** Correlation between synovial markers and serum metabolites. (TIFF 14826 kb)

Additional file 3: **Figure S2.** Pathway analysis of polar compounds by MetaboAnalyst. (TIFF 14826 kb)

Additional file 4: **Figure S3.** Correlation between serum metabolites and synovial CD19, CD79A, and IgGHC. (TIFF 14826 kb)

Additional file 5: **Figure S4.** Correlation between serum metabolites and synovial APRIL, CD138, SDF1, IgKappa, and IgMHC. (TIFF 14826 kb)

Additional file 6: **Figure S5.** Correlation between serum metabolites and synovial MMP1, MMP3, and IL-6. (TIFF 14826 kb)

Additional file 7: **Figure S6.** Correlation between serum metabolites and synovial IL-1β and IL-8. (TIFF 14826 kb)

Additional file 8: **Figure S7.** Correlation between serum cytokines and synovial cytokines and serum metabolites. (TIFF 14826 kb)

## Abbreviations

APRIL: A proliferation-inducing ligand
ARISE: Assessment of rituximab’s immunomodulatory synovial effects
ATP: Adenosine triphosphate
BLySB: Lymphocyte stimulator
CRP: C-reactive protein
CTL: Choline like transporter
DAS: Disease activity score
FLS: Fibroblast-like synoviocytes
HAQ: Health assessment questionnaire
HIF-1α: Hypoxia-inducible factor-1α
IL-1β: Interleukin
MMP: Matrix metalloproteinase
MTX: Methotrexate
NMR: ^1^H-nuclear magnetic resonance
RA: Rheumatoid arthritis
RF: Rheumatoid factor
SDF1: S cell-derived factor 1
TCA: Tricarboxylic acid
TMA: Trimethylamine
TMAO: Trimethylamine N-oxide
TNF-α: Tumor necrosis factor
