# Supplementary material for: Serum metabolomic profiling predicts synovial gene expression in rheumatoid arthritis
Source: Arthritis Res Ther. 2018 Aug 3;20:164. doi: 10.1186/s13075-018-1655-3 (PMC6091066; doi:10.1186/s13075-018-1655-3)
Supplement: Supplementary file 1 — Table S1. Baseline clinical characteristics of patients with rheumatoid arthritis. Table S2. Mean and standard deviation (SD) of synovial biomarker expression. Table S3. Mean and standard deviation (SD) of serum metabolites detected by 1H-NMR (μM). Reference values are from the Human Metabolome Database (HMDB) and were collected via NMR, unless otherwise noted. 1GC/MS; 2HPLC; 3HPLC-fluoroescence; 4ion-exchange chromatography; 5DFI/MS/MS 6unknown. ND, no data available. Metabolites that were upregulated by at least 20% compared to reference values are in green. Metabolites that were downregulated by more than 20% compared to reference values are in red. (DOCX 26 kb) [file 13075_2018_1655_MOESM1_ESM.docx]

**SUPPLEMENTARY INFORMATION**

Figure S1: **Correlation of synovial markers with serum metabolites.** Linear regression was performed between each synovial marker – serum metabolite pair, controlling for age and gender. This figure displays metabolite regression adjusted p-values after applying Benjamini-Hochberg false discovery rate to correct for multiple testing. Row and column order are preserved from Figure 3A.

Figure S2: **Pathway analysis of polar compounds by MetaboAnalyst**. Pathway p-values are calculated based on metabolites that correlate with different cytokine clusters.

Figure S3: **Correlation of serum metabolites with synovial CD19, CD79A and IgGHC.**  A) Correlation of serum metabolites for each synovial marker, using linear regression, and controlling for both age and gender. We also included the Benjamini-Hochberg false discovery rate adjusted p-values to correct for multiple testing. B) Overview of the metabolites identified by NMR, organized by metabolic pathway. In red are metabolites that negatively correlate with CD19, CD79A and IgGHC. Abbreviations: TMA: trimethylamine, TMAO: trimethylamine N-oxide, DMA: NN-dimethylamine, THF: Tetrahydrofolate, IMP: inosine monophosphate. C) Pathway analysis of polar compounds by MetaboAnalyst. Pathway p-values are calculated based on metabolites that correlate with CD19, CD79A and IgGHC.

Figure S4: **Correlation of serum metabolites with synovial APRIL, CD138, SDF1, IgKappa and IgMHC.**  A) Correlation of serum metabolites for each synovial marker, using linear regression, and controlling for both age and gender. We also included the Benjamini-Hochberg false discovery rate adjusted p-values to correct for multiple testing. B) Overview of the metabolites identified by NMR, organized by metabolic pathway. In red are metabolites that negatively correlated with APRIL, CD138, SDF1, IgKappa and IgMHC. Abbreviations: TMA: trimethylamine, TMAO: trimethylamine N-oxide, DMA: NN-dimethylamine, THF: Tetrahydrofolate, IMP: inosine monophosphate. C) Pathway analysis of polar compounds by MetaboAnalyst. Pathway p-values are calculated based on metabolites that correlate with APRIL, CD138, SDF1, IgKappa and IgMHC.

Figure S5: **Correlation of serum metabolites with synovial MMP1, MMP3 and IL-6.**  A) Correlation of serum metabolites for MMP1, MMP3 and IL-6, using linear regression, and controlling for both age and gender. We also included the Benjamini-Hochberg false discovery rate adjusted p-values to correct for multiple testing. B) Overview of the metabolites identified by NMR, organized by metabolic pathway. In red are metabolites that negatively correlated with MMP1, MMP3 and IL-6. Abbreviations: TMA: trimethylamine, TMAO: trimethylamine N-oxide, DMA: NN-dimethylamine, THF: Tetrahydrofolate, IMP: inosine monophosphate. C) Pathway analysis of polar compounds by MetaboAnalyst. Pathway p-values are calculated based on metabolites that correlate with MMP1, MMP3 and IL-6.

Figure S6: **Correlation of serum metabolites with synovial IL-1β and IL-8.**  A) Correlation of serum metabolites for IL-1β and IL-8, using linear regression, and controlling for both age and gender. We also included the Benjamini-Hochberg false discovery rate adjusted p-values to correct for multiple testing. B) Overview of the metabolites identified by NMR organized by metabolic pathway. In red are metabolites that negatively correlated with IL-1β and IL-8, and in green are metabolites that positively correlated with them. Abbreviations: TMA: trimethylamine, TMAO: trimethylamine N-oxide, DMA: NN-dimethylamine, THF: Tetrahydrofolate, IMP: inosine monophosphate. C) Pathway analysis of polar compounds by MetaboAnalyst. Pathway p-values are calculated based on metabolites that correlate with IL-1β and IL-8.

Figure S7: **Correlation of serum cytokines with synovial cytokines and serum metabolites.** A) Linear regression was performed between each serum cytokine-synovial cytokine. We included a Benjamini-Hochberg false discovery rate adjusted p-values to correct for multiple testing. The regression coefficients for each pair were used to form a clustered heatmap. None of the corresponding p-values reached statistically significance. B) Linear regression was performed between each serum cytokine – serum metabolite pair, controlling for age and gender. Right: The regression coefficients for each pair were used to form a clustered heatmap, to lend insight into which serum cytokine correlated with which serum metabolite. Row clusters have been identified by cophenetic cutting of the row dendrogram. Left: Metabolite regression p-values are displayed in left, where the row and column order are preserved from right.

| Characteristic | Mean (Std. Dev) |
| --- | --- |
| Female (%)  Age | 74  52.7(12.1) |
| Duration RA (Years) | 11.5 (7.6) |
| ESR | 47.3(33) |
| CRP | 13.4(23) |
| Pain | 64.6(23.9) |
| Patient Global | 64 (27.4) |
| MD Global | 59.2(29.2) |
| HAQ | 1.9(0.69) |
| DAS | 6.4(1.1) |
| Tender Joints (28) | 16.1(8.5) |
| Swollen Joints (28) | 14.1(8.2) |
| CCP screening | 127.9(167.2) |

**Table S1.** Baseline clinical characteristics of rheumatoid arthritis patients

**Table S2.** Mean and standard deviation (SD) of synovial biomarker expression.

| \| Biomarker \| Mean (SD) \| \| --- \| --- \| \| TNF-a \| 0.219 (0.075) \| \| IL-1β \| 0.011 (0.016) \| \| IL-6 \| 0.539 (0.902) \| \| IL-8 \| 0.046 (0.108) \| \| MMP1 \| 1.027 (2.082) \| \| MMP3 \| 2.580 (5.153) \| \| CD19 \| 0.011 (0.019) \| \| CD79A \| 0.029 (0.045) \| \| CD138 \| 0.049 (0.054) \| \| IgMHC \| 0.22 (0.393) \| \| IgG1HC \| 0.003 (0.006) \| \| IgKappa \| 0.479 (0.830) \| \| BLy-S \| 2.126 (1.915) \| \| SDF1 \| 1.138 (1.071) \| \| APRIL \| 5.047 (3.46) \| \| CD3E \| 0.066 (0.068) \| |  |  |  |
| --- | --- | --- | --- | --- | --- | --- | --- | --- | --- | --- | --- | --- | --- | --- | --- | --- | --- | --- | --- | --- | --- | --- | --- | --- | --- | --- | --- | --- | --- | --- | --- | --- | --- | --- | --- | --- | --- |

**Table S3.** Mean and standard deviation (SD) of serum metabolites detected by 1H-NMR (μM). Reference values are from the Human Metabolome Database (HMDB) and were collected via NMR, unless otherwise noted. ^1^GC/MS ^2^HPLC ^3^HPLC-Fluoroescence ^4^Ion-exchange chromatography ^5^DFI/MS/MS ^6^Unknown. ND: No data available. Metabolites that were upregulated at least 20% compared to reference values are in green. Metabolites that were downregulated more than 20% compared to reference values are in red.

| Metabolite | Mean (SD) Normal range (SD) | |
| --- | --- | --- |
| Oxoisocaproate | 8.482 (2.909) | 28 (0-58)^1^ |
| Acetone | 16.405 (9.809) | 54.4 (29.6) |
| Alanine | 362.667 (127.246) | 427.2 (84.4) |
| Aspartate | 76.702 (38.584) | 20.9 (6.1) |
| Azelate | 79.896 (58.672) | 27 (0-58)^1^ |
| Betaine | 58.067 (18.163) | 72 (22.4) |
| Carnitine | 26.693 (9.167) | 45.7 (11.6) |
| Choline | 9.402 (4.168) | 14.5 (5.3) |
| Citrate | 115.886 (40.507) | 114.2 (27) |
| Creatine | 25.248 (13.580) | 37.6 (28.3) |
| Creatinine | 58.199 (17.712) | 88.6 (18.8) |
| Dimethylsulfone | 6.202 (16.578) | 8.8 (7.3) |
| Formate | 21.295 (8.367) | 32.8 (13.3) |
| Glucose | 3780.422 (1518.272) | 4971.3 (378.2) |
| Glutamate | 182.018 (82.201) | 97.4 (13.2) |
| Glutamine | 508.611 (149.510) | 510.4 (118.2) |
| Glycine | 262.499 (83.608) | 325.4 (126.8) |
| Isoleucine | 59.670 (19.137) | 60.7 (18.6) |
| Lactate | 3993.232 (3210.518) | 1489.4 (371.2) |
| Leucine | 151.721 (44.72) | 98.7 (11.5) |
| Lysine | 134.175 (36.734) | 178.6 (58.2) |
| Methionine | 19.349 (6.363) | 29.8 (6.3) |
| Methylmalonate | 23.807 (15.477) | 0.187 (0.084)^3^ |
| Methylsuccinate | 12.997 (3.517) | ND |
| Ornithine | 52.323 (27.770) | 66.9 (15.3) |
| Pantothenate | 2.513 (0.868) | 4.91 (0.38)^6^ |
| Phenylalanine | 69.493 (24.094) | 78.1 (20.5) |
| Propyleneglycol | 172.361 (131.847) | 22.3 (3.3) |
| Pyruvate | 64.542 (41.240) | 34.5 (25.2) |
| Serine | 163.888 (46.628) | 159.8 (26.6) |
| Succinate | 9.734 (5.375) | 23.5 (16.0)^1^ |
| Threonine | 342.136 (418.844) | 127.7 (41) |
| Trimethylamine | 1.024 (0.682) | 0.418 (0.124)^1^ |
| Tyrosine | 62.747 (20.130) | 54.5 (9.7) |
| Urea | 5786.740 (2072.884) | 6074.6 (2154.2) |
| Valine | 212.869 (62.935) | 212.3 (61.3) |
| Xanthine | 6.693 (3.334) | 1.27 (0.87) |
| betaAlanine | 2.633 (1.059) | 3.8 (2.9)^2^ |
| Fumarate | 1.030 (0.917) | 1.5 (0-4)^1^ |
| 3-Hydroxybutyrate | 104.990 (82.427) | 76.9 (66.3) |
| N,N-dimethylamine | 1.657 (0.480) | 48.35 (7.3)^2^ |
| O-acetylcarnitine | 4.192 (1.568)^5^ | 5.476 (2.147)^2^ |
| Acetoacetate | 12.734 (7.691) | 40.6 (36.5) |
| Taurine | 61.434 (123.623) | 55 (13)^4^ |
| Hypoxanthine | 17.271 (14.925) | 34.2 (10.3) |
| 2-Hydroxyvalerate | 7.010 (6.975) | ND |
| Asparagine | 26.930 (7.946) | 82.4 (7.3) |
| Glycerol | 81.523 (35.271) | 431.6 (100.3) |
| Trimethylamine N-oxide | 38.508 (16.876) | 37.8 (20.4) |
