# Supplementary figures and images for: Serum metabolomic profiling predicts synovial gene expression in rheumatoid arthritis
Source: Arthritis Res Ther. 2018 Aug 3;20:164. doi: 10.1186/s13075-018-1655-3 (PMC6091066; doi:10.1186/s13075-018-1655-3)

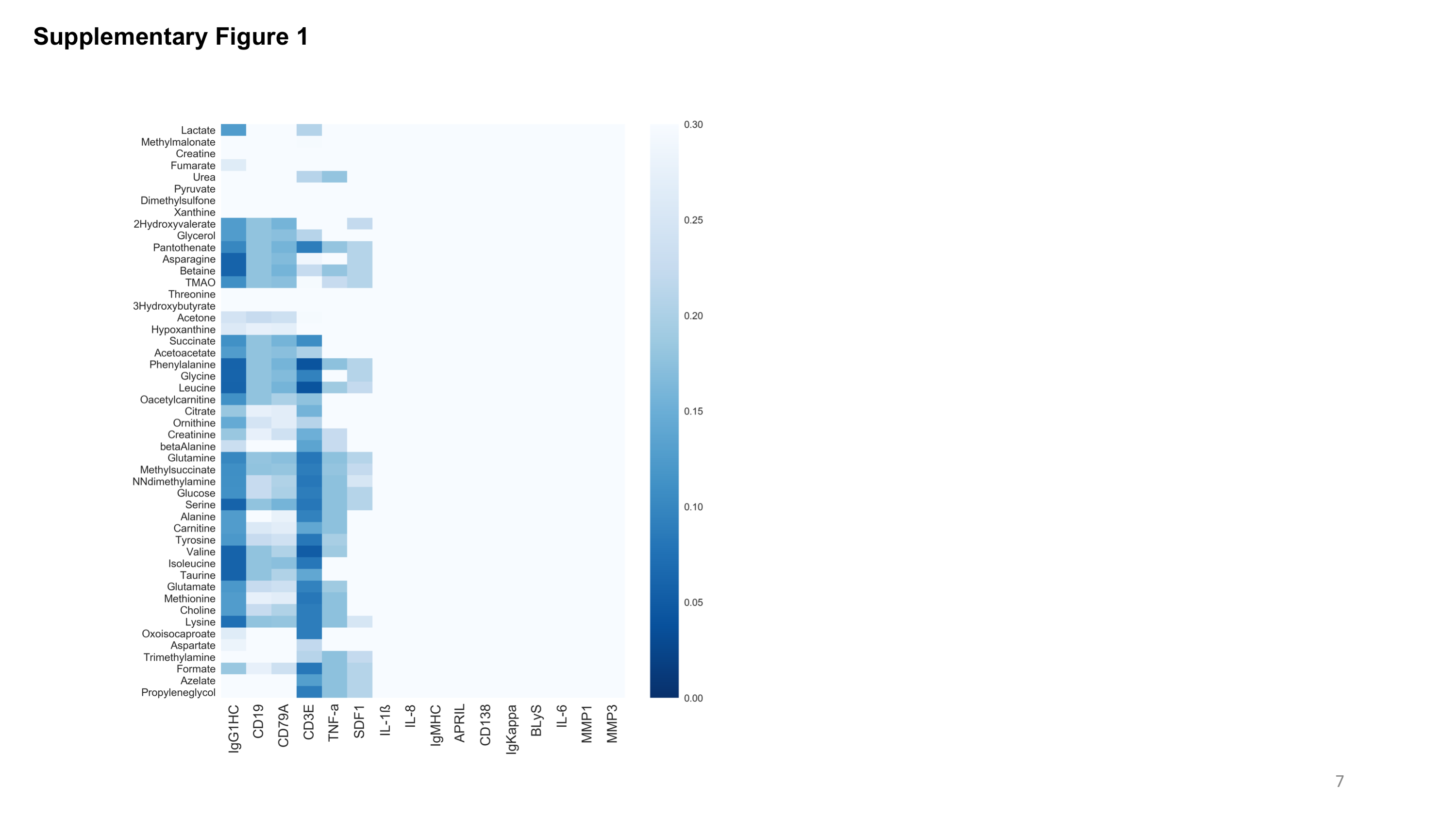

Supplement: Supplementary file 2 — Figure S1. Correlation between synovial markers and serum metabolites. (TIFF 14826 kb) [file 13075_2018_1655_MOESM2_ESM.tiff]

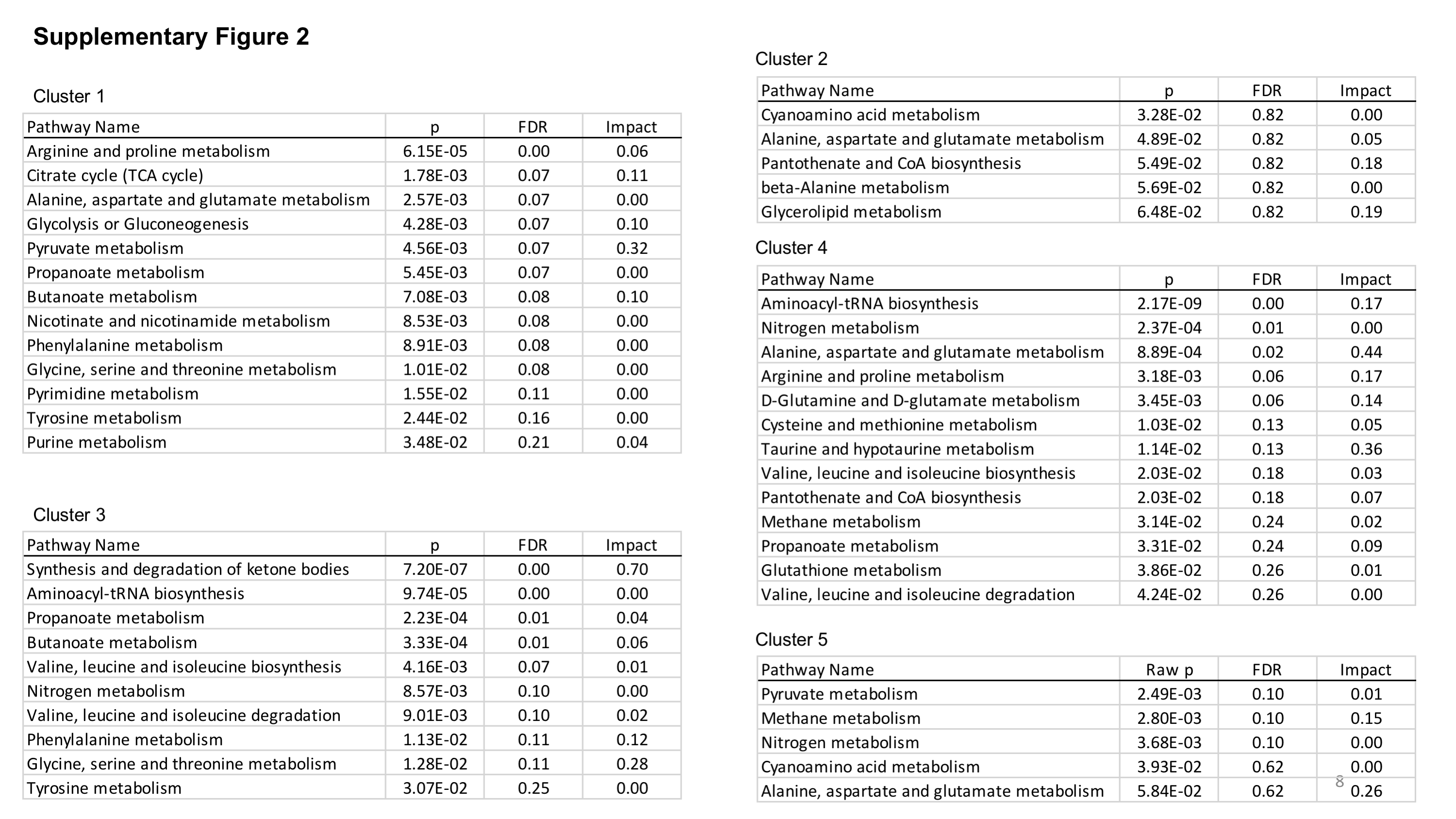

Supplement: Supplementary file 3 — Figure S2. Pathway analysis of polar compounds by MetaboAnalyst. (TIFF 14826 kb) [file 13075_2018_1655_MOESM3_ESM.tiff]

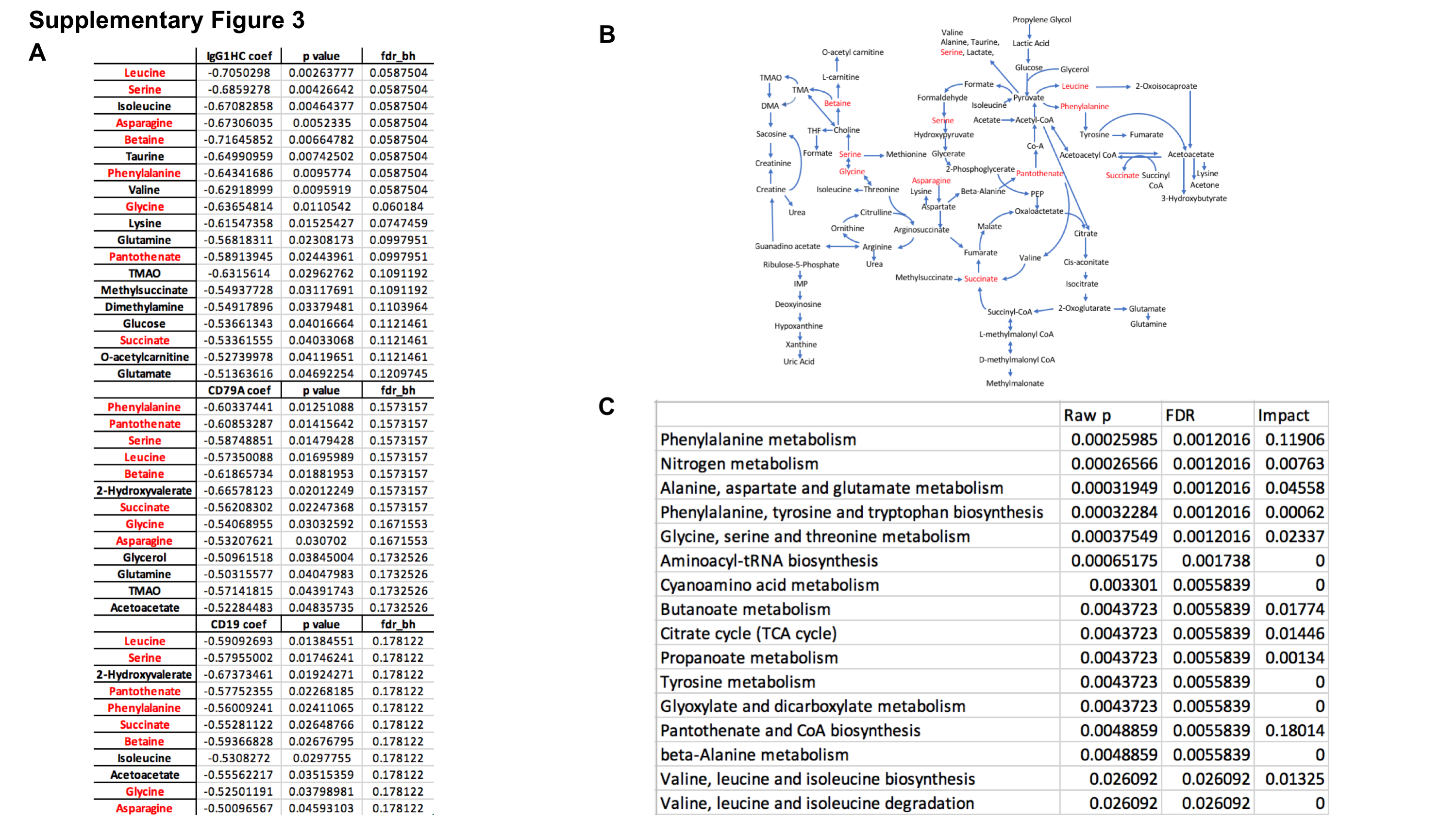

Supplement: Supplementary file 4 — Figure S3. Correlation between serum metabolites and synovial CD19, CD79A, and IgGHC. (TIFF 14826 kb) [file 13075_2018_1655_MOESM4_ESM.tiff]

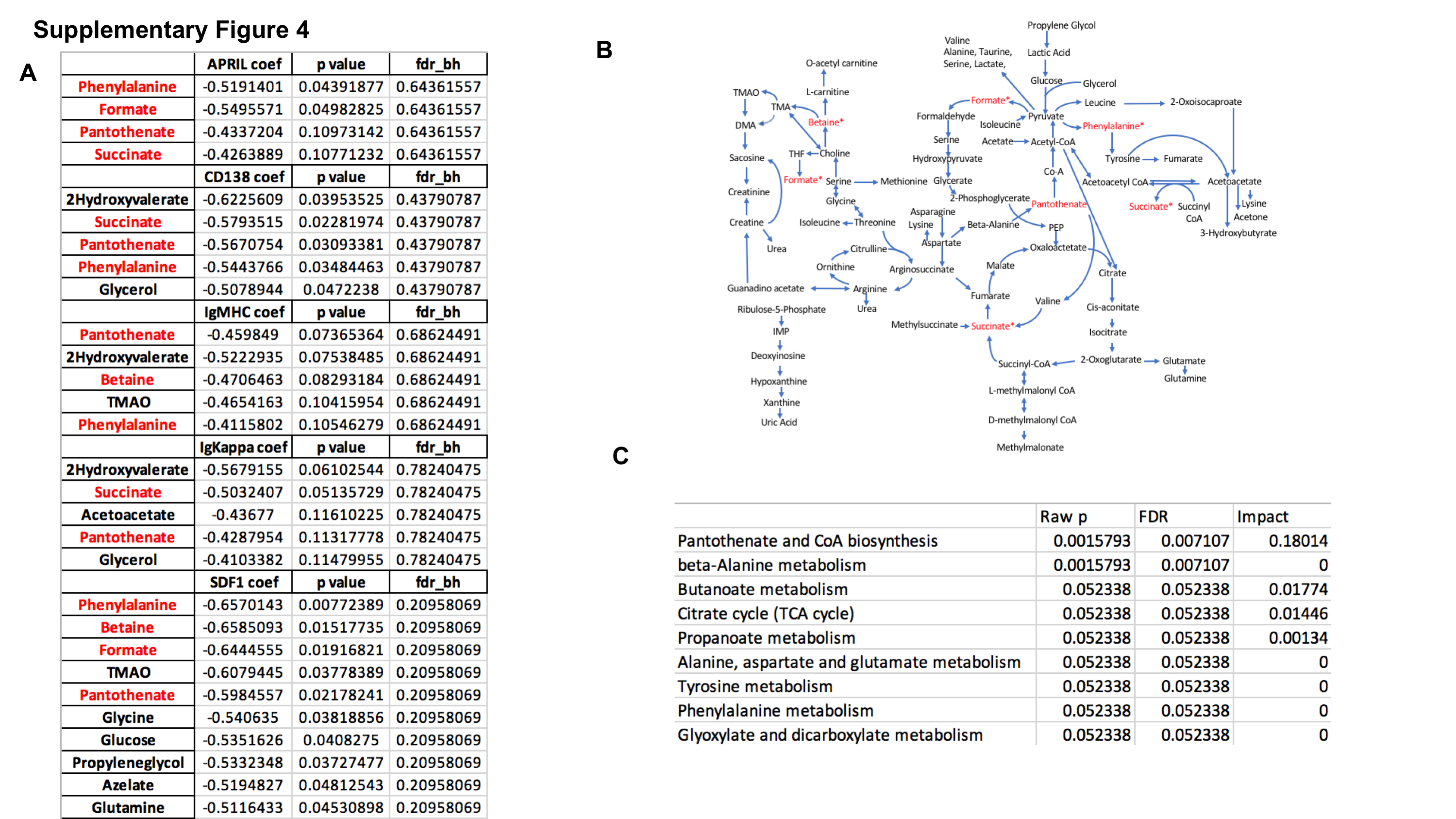

Supplement: Supplementary file 5 — Figure S4. Correlation between serum metabolites and synovial APRIL, CD138, SDF1, IgKappa, and IgMHC. (TIFF 14826 kb) [file 13075_2018_1655_MOESM5_ESM.tiff]

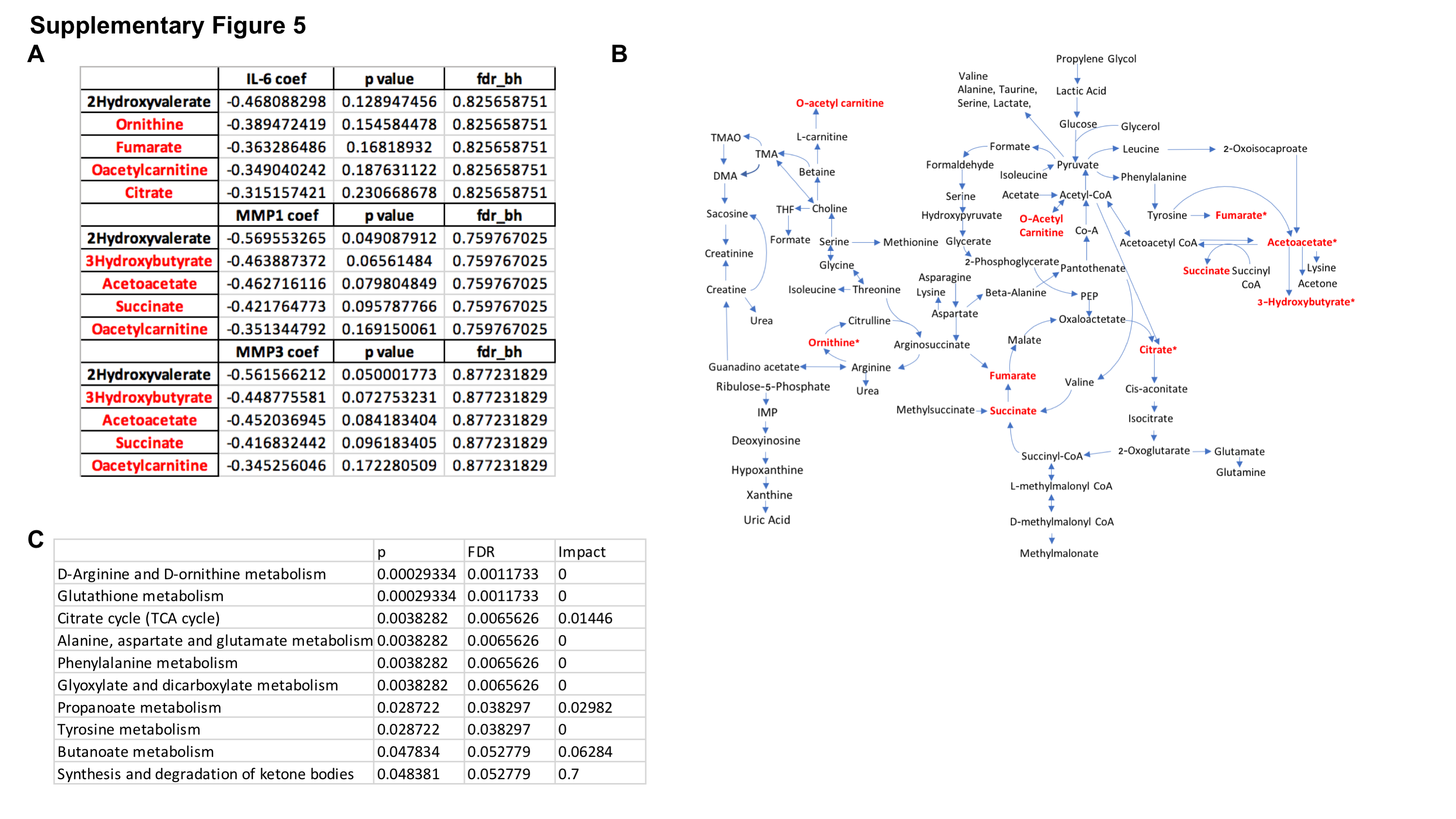

Supplement: Supplementary file 6 — Figure S5. Correlation between serum metabolites and synovial MMP1, MMP3, and IL-6. (TIFF 14826 kb) [file 13075_2018_1655_MOESM6_ESM.tiff]

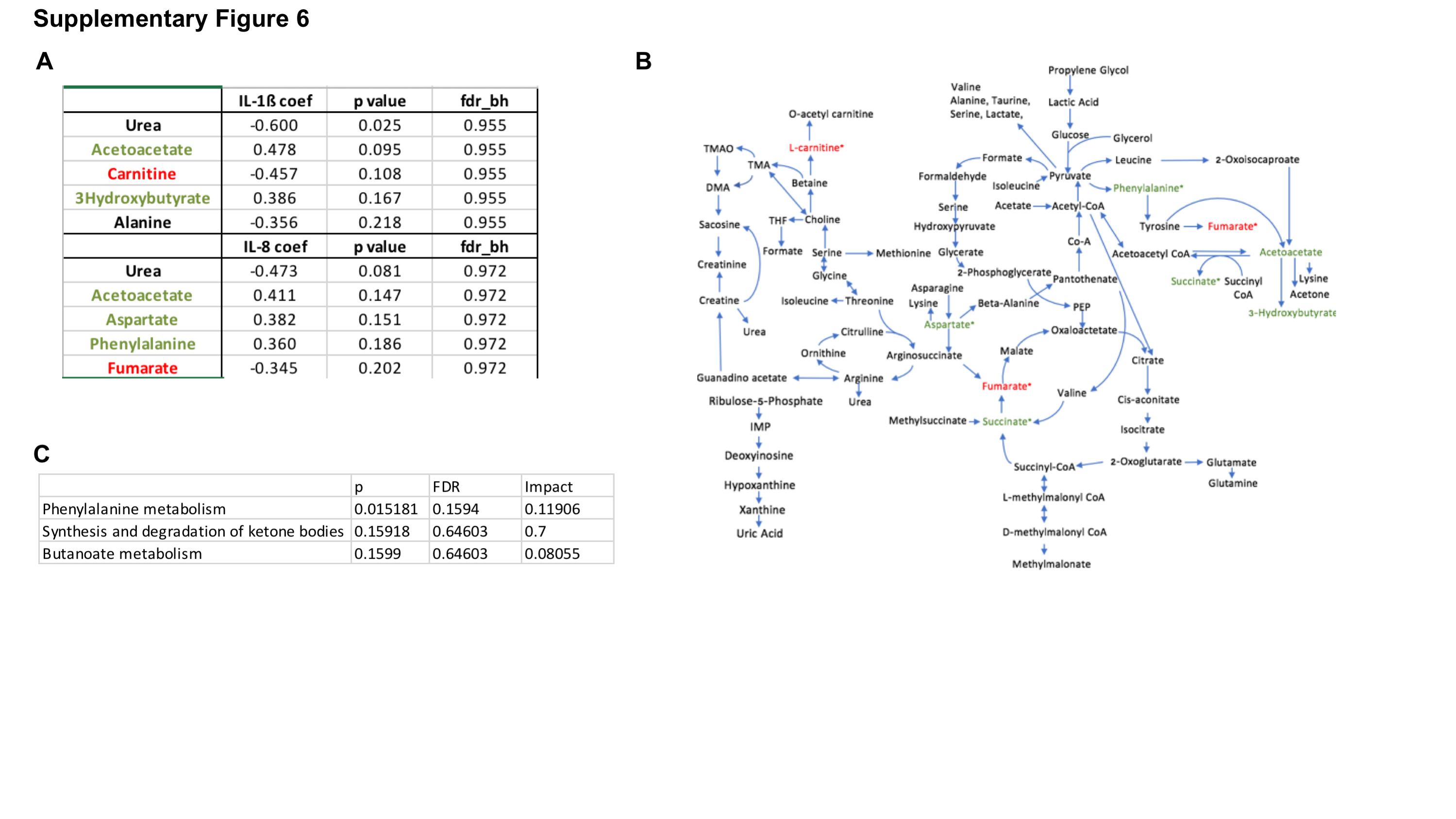

Supplement: Supplementary file 7 — Figure S6. Correlation between serum metabolites and synovial IL-1β and IL-8. (TIFF 14826 kb) [file 13075_2018_1655_MOESM7_ESM.tiff]

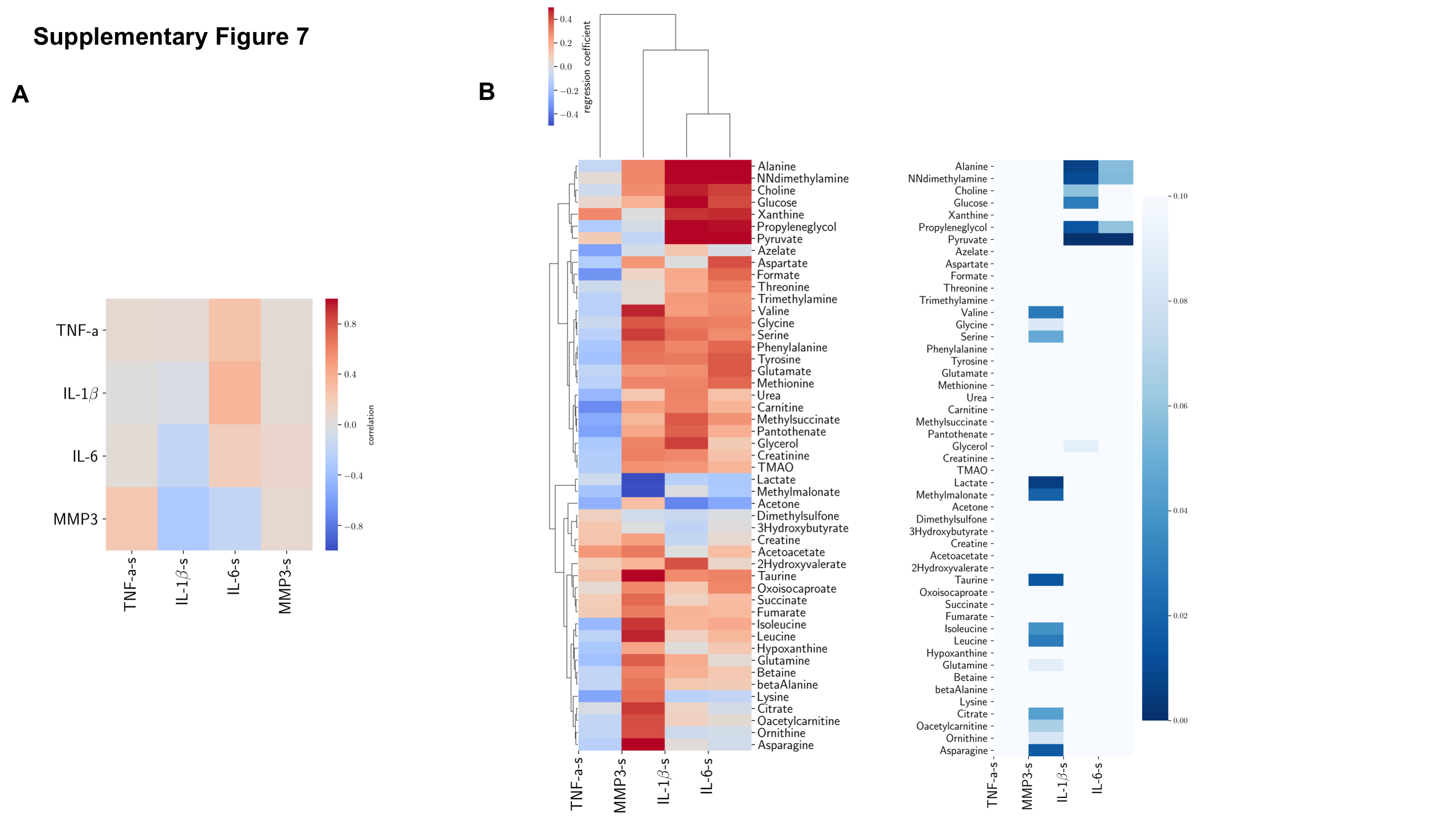

Supplement: Supplementary file 8 — Figure S7. Correlation between serum cytokines and synovial cytokines and serum metabolites. (TIFF 14826 kb) [file 13075_2018_1655_MOESM8_ESM.tiff]
